# Neutrophil extracellular trap-mediated activation of endosomal toll-like receptors induce immune activation in juvenile-onset systemic lupus erythematosus

**DOI:** 10.1186/1546-0096-12-S1-P110

**Published:** 2014-09-17

**Authors:** Kanchani K Makuloluwa, Angela Midgley, Michael Beresford

**Affiliations:** 1 Institute of Child Health, University of Liverpool, Liverpool, UK

## Introduction

Juvenile-onset Systemic Lupus Erythematosus (JSLE) is a multisystem autoimmune disorder characterized by the overproduction of autoantibodies against nuclear self-antigens. Activated neutrophils may undergo cell death by NETosis, forming mesh-like structures termed Neutrophil extracellular traps (NETs). Comprising of DNA, histones and antimicrobial proteins, increasing evidence implicates NETs in many pathological conditions, including SLE. Toll like receptors (TLRs) are pattern recognition receptors capable of mediating immune responses. Endosomally localised TLR 7 and 9, expressed highly in JSLE, can detect nuclear antigens. Our hypothesis is that NETs offer a novel source of nuclear auto-antigens that are being detected via TLR 7/9, resulting in the downstream activation of the immune system.

## Objectives

To investigate whether NETs originating from JSLE neutrophils are providing a source of auto-antigens detected through TLRs and whether they are a determining factor in the activation of the immune system.

## Methods

Neutrophils isolated from JSLE and paediatric control donors were incubated with interferon-alpha (IFN-a) and JSLE sera for 2 hours to induce NET formation. NETs were subsequently treated with Micrococcal nuclease to dismantle NET scaffold, leaving a cell-free supernatant of NET material comprising of double stranded DNA, which was quantified using Quan-iT Picogreen Assay. Peripheral blood mononuclear cells (PBMCs) isolated from healthy adult controls were incubated for 15 minutes with a known concentration of NET dsDNA derived from JSLE (n=5) and paediatric control (n=5) neutrophils. Cell protein was extracted and pIRAK1 (a downstream signalling protein of TLR7 and 9) expression was determined by western blot analysis, normalised to B-actin expression. NETs were also incubated with PBMCs pre-treated with hydroxychloroquine, and subsequent pIRAK1 expression was determined by western blot analysis. Hydroxychloroquine was added to inhibit endosomal TLR7/9 activation, and to confirm whether NET induced stimulation was TLR dependent.

## Results

PBMCs incubated with IFN-a-induced NETs showed increased pIRAK1 protein expression (arbitrary units; Mean ±SEM) in both JSLE (0.54±0.14, n=5) and paediatric control (0.59±0.15, n=5) neutrophil-derived NETs, as compared to unstimulated PBMCs (0.21). The increased pIRAK1 expression was not influenced by the source of the NETs for IFN-a induced NETs. However, preliminary data for JSLE sera induced NETs indicate that the origin of the NETs may influence pIRAK1 expression, with JSLE neutrophils inducing increased TLR activation (1.66±0.50, n=5) compared to control neutrophils (0.71±0.22, n=5) and unstimulated PBMCs (0.29). Incubation of NETs with PBMCs pre-treated with hydroxychloroquine reduced pIRAK1 expression in a dose-dependent manner, indicating that NETs have activated the immune system via TLR 7 and 9.

## Conclusion

We have demonstrated that IFN-a and JSLE serum-induced NETs may be an important source of auto antigens in JSLE that are being detected through TLR 7/9 leading to the activation of these receptors. Furthermore, inhibition of TLR7/9 signalling reduced NET mediated activation of these receptors. Further studies are warranted of this important pathway in JSLE in order to identify potential therapeutic targets.

## Disclosure of interest

None declared.

